# Trust in Medical AI: The Case of mHealth Diabetes Apps

**DOI:** 10.1111/jep.70216

**Published:** 2025-08-13

**Authors:** Sophie Materne, Stefano Canali, Daniele Chiffi

**Affiliations:** ^1^ Department of Electronics Information and Bioengineering, Politecnico di Milano Milan Italy; ^2^ DAStU Politecnico di Milano Milan Italy

**Keywords:** diabetes, ethics of AI, medical AI, mHealth, risk, trust, trustworthy AI

## Abstract

Over the past few years, mobile health applications (mHealth apps) have gained popularity regarding the management of chronic conditions, such as diabetes, as they are considered to enhance follow‐up and treatment. Indeed, these applications are powerful tools that support individualised pharmaceutical and non‐pharmaceutical care by remotely monitoring the patient's health status in real‐time. Nevertheless, concerns have been raised regarding trust and trustworthiness towards their use, in particular, when they are powered by Artificial Intelligence (AI). Trust in AI is a multifaceted notion that encompasses aspects such as reliance, risk, as well as ethical principles (e.g., respect for human autonomy and prevention of harm). In this paper, we provide an analysis of trust and trustworthiness in AI‐powered mHealth apps for diabetes through the lens of philosophy and risk analysis to promote the patient's well‐being.

## 1 Introduction

Healthcare systems are currently undergoing a profound digital transformation with, amongst other things, the emergence of mobile health applications either based on Artificial Intelligence (AI)^1^ or having AI components. One of the most significant promises of the use of AI for the medical sector is the self‐management of chronic diseases, such as diabetes [3]. By being available on personal mobile devices and, therefore, directly in contact with the patient, health applications that run on smartphones—known as mHealth apps—are becoming a crucial tool to allow users to monitor their health condition effectively, continuously, and remotely. These technologies can provide users with tools to proactively manage their health [4]. In this way, mHealth apps are often assumed to promote patient empowerment [5], shifting many aspects of health management into the hands of patients [6]. These apps capture a wide range of clinical and nonclinical patient data to further provide tailored information, which may result in an overall life quality improvement.

Although mHealth apps seem to be promising in facilitating numerous aspects of conditions such as diabetes [7, 8], various issues have emerged and have been discussed in the literature. For instance, the literature has looked at issues pertaining to privacy and security, inadequate responsiveness to urgent situations, challenges for users in evaluating the app's content quality, and user acceptance [9, 10, 11]. We hold the view that many of these concerns can be seen as issues of trust and trustworthiness, and an elucidation of trust and trustworthiness in the context of mHealth apps may be beneficial to ensure benefits, prevent harmful implications, and improve their effectiveness. In this paper, we provide a critical review of current research and cases of diabetes mHealth apps exploiting AI, as well as an analysis of this issue of trust in this context based on recent work on trust and AI and its connection to reliance, risk, and ethical considerations [12].

The notion of trust is particularly relevant in the context of mHealth apps as it is considered a crucial factor for the social acceptability and general use of the technology [13, 14]. The fact that these technologies make use of AI makes the issue of trust particularly complex, given the common opacity and lack of explainability usually associated with AI models [15, 16]. A conceptual elucidation of the notion of trust, conducted through the lens of epistemology and ethics, is thus crucial for the context of mHealth apps, where trust is particularly connected to many benefits for the patients but also potentially high risks.

We will focus in particular on the context of mHealth apps for the treatment of diabetes, a chronic condition (chronic disease management is one of the most dominant areas for mHealth app usage), where patient trust is a crucial element [17]. This focus enables us to capture the ethically relevant and value‐laden aspects of the notion of trust in association with an analysis of a variety of different risks. Linking theoretical foundations to real‐world experiences in the context of mHealth apps for diabetes has recently been acknowledged as a crucial point for developing effective and user‐centred interventions [18].

The paper is structured as follows. We first highlight the main patient‐specific features of the management of diabetes and discuss the current promises and challenges of diabetes mHealth apps exploiting AI in Section 2. In Section 3, we provide an overview of some of the main philosophical perspectives of trust and trustworthiness in medical AI, and then we elaborate a framework for these concepts based on reliance, risk, and ethically relevant values. On this ground, we show the application of this framework to diabetes mHealth apps in Section 4. Finally, Section 5 concludes the paper, discussing further lines of research and theoretical approaches that may be usefully adopted in the context of mHealth apps.

## 2 Diabetes Management and the Rise of mHealth Apps

Diabetes is a chronic and metabolic disease characterised by raised blood sugar concentration (hyperglycaemia) caused either by deficient insulin production (type I diabetes) or by insulin resistance (type II diabetes). As a hypoglycaemic hormone, the function of insulin is to lower the amount of blood glucose to bring it back to a safe level. Diabetes alters the body's ability to properly regulate its blood sugar level and can lead to various severe or mild short‐ and long‐term impairments when uncontrolled or poorly managed. Short‐term impacts encompass symptoms such as excessive thirst, frequent need to urinate, blurred vision and headaches as well as weakness, tiredness and shakiness. Long‐term consequences include morbidities such as cardiovascular disease, strokes, kidney failures (nephropathy), eye problems (retinopathy), foot injuries (ulcers, infections, and amputation), as well as nerve damage (neuropathy). If diabetes is not correctly treated, these complications can contribute significantly to poor quality of life, financial burdens to both patients and society, and even mortality [19]. At the same time, the management of diabetes needs to be patient‐specific and consider individual features of patients, as well as its type and severity. Daily consistent insulin injections are required in the case of type I, while type II is mainly managed based on lifestyle interventions and changes (diet and physical activity).

To achieve personalised optimal glycaemic control, and thus prevent or reducing associated side‐effects, continuous monitoring of glucose level is crucial, and this is where apps based on AI are increasingly used and can be helpful, especially for type I diabetes. These apps provide a variety of features, including blood glucose level tracking, insulin dosages, diet monitoring, and education provision [20, 21]. They also offer a wide range of customised interacting functionalities and features, which varies across different apps and types of diabetes targeted by the app. These include, amongst others, glucose monitoring through integrated blood glucose metres, medication adherence tracking, nutrition and fitness activity planning, data collection, processing, and analysis, notifications (reminders and alerts), communication and data exchange with healthcare professionals (HCPs), educational resources as well as community and psychological support [19, 22].^2^

The number of commercialised diabetes mHealth apps has been increasing in the last decade. The U.S. Food and Drug Administration (FDA) certified the Guardian Connect System in 2018 as the first smart continuous glucose monitoring coupled to a smartphone app [21]. This app makes use of AI at its core through a predictive algorithm that aims to predict hypo‐ and hyperglycaemic events in patients with type I or II diabetes. Two years later, CampAPS FX^3^ was launched as the world's first licensed, downloadable artificial pancreas app for people with type I diabetes in the UK. This app employs AI modelling connected to an insulin pump and a glucose monitor to continuously detect low glucose levels and then automatically administer the optimal insulin dose thanks to its adaptive algorithm.

We can draw a few distinctions concerning the functions of these apps and their use of AI models. A first distinction can be made between general diabetes mHealth apps for self‐monitoring and those specifically designed for continuous glucose monitoring. The former category is a subset of the latter and can be described as advanced diabetic diaries [22], which are useful but not necessary for glucose monitoring and managing diabetes. These apps mainly use AI for secondary options (e.g., ESYSTA, that is designed for both types of diabetes) and tend to allow users to log blood sugar levels, track medication, and monitor dietary habits, but usually lack the integration with continuous glucose monitoring systems.^4^ As such, general diabetes mHealth apps can be differentiated from apps designed for continuous diabetes monitoring, that are typically bundled with glucose sensors and offer a range of features tailored to real‐time management of diabetes. For example, popular continuous glucose‐monitoring systems, such as Dexcom, come with dedicated applications that provide users with access to their glucose levels without the need for a fingerstick.^5^ These apps display current glucose readings but also include features like meal logging, activity tracking, motivation, alerts for high or low glucose levels, trends over time, and data summaries that can be shared with HCPs. This integration allows for a more proactive approach to diabetes management, enabling users to make decisions based on real‐time data, often supported by AI‐based models.

A second distinction we can draw concerns type I and type II diabetes. While some apps are presented as approaches to both types, type I diabetes mHealth apps have a higher use of AI modelling at their core and we will mostly focus our analyses on these as a result. AI is often embedded in type I diabetes mHealth apps to enable advanced features, such as glucose level forecasting and insulin dose adjustments. In the literature, their features have been categorised into six classes: self‐care data monitoring, app display, feedback and reminders, data entry, data sharing, and additional features [19]. The factors affecting the use of these apps have also been identified as being related to personal factors, privacy and safety factors, socioeconomic aspects, as well as app design or usability. Regarding the latter, it is worth noting that it can be measured by the attributes that are the most used ones according to the ISO9241‐11 usability definition: efficiency, satisfaction, and effectiveness; other attributes can be considered too, such as learnability, memorability, cognitive load, errors, simplicity, and ease of use [20].

Hence, several mHealth apps are available for diabetes management and incorporate AI. An emerging literature has started to pay more attention to these apps and their benefits and limitations. The main investigated aspects linked to those apps are the design features [19, 20, 21, 23], the clinical effectiveness, and the patient‐specific perception [21, 23, 24]. These are all aspects that should be examined from a trust perspective, but to the best of our knowledge the concept of Trustworthy AI (TAI) and its relation to diabetes mHealth apps has not been investigated yet. Our work aims to fill this gap by first developing the notion of trust in AI in general and then specifically applying it to diabetes mHealth apps.

## 3 Trust in AI: A Conceptual Framework

Our increasing dependence on AI systems has raised several questions about the potential repercussions of trust in technology in recent years [25, 26, 27, 28]. Trust has been considered useful to manage the risks associated with specific AI uses in the context of several regulations and guidelines. These guidelines aim to promote the trustworthy development of AI systems grounded in responsible and socially conscious practices [29, 30].^6^ These sets of general recommendations and/or obligations, regarding the different agents involved at any stage of the development, provision, maintenance, and even use of the AI technology have been assumed to be necessary prerequisites for the users to be able to put their trust in the tasks the systems are supposed to complete [31]. More specifically, the FDA has recently introduced a framework about AI‐driven medical products, aimed at promoting a rapid cycle of product enhancement. This framework allows these medical products to continually refine their functionalities while ensuring stringent protective measures are upheld, even in the pre‐market stage, to reduce potential risks [32].

Particular attention has been devoted to the concepts of trust in AI and TAI in the philosophical literature, as the use of AI artefacts grows in scope with transformative implications, shaping users' understanding and identity through social‐technological activity [25, 33, 34].^7^ Also, AI systems are artefacts that ‘mediate our social and cultural, but also economic and political interactions’ [36] and are increasingly and extensively leveraged in a wide range of products and services spanning various domains of daily life, including medicine, justice systems and financial markets. In addition, the fact that they are often based on opaque algorithms raises concerns about trust, as the internal mechanisms transforming the input(s) into the output(s) may not be accessible and understandable to humans [37]. Thus, worries regarding those opaque algorithms have led to ethical concerns on partiality, autonomy and responsibility, amongst others [38]. However, well‐explainable statistical and machine learning methods for predicting low glucose levels are available [39].

Trust in AI is a multifaceted notion approached and discussed in the literature from different angles with different terminologies, with various theories of trust in AI emerging as a result [40, 41]. In addition to reliance, on which it is sometimes grounded and that refers to the accuracy and completeness of data (e.g., absence of data errors, duplicates, missing values, etc.) and expresses the practical dependence on AI to perform a task [42], trust has been discussed in relation to other dimensions that can be categorised into human factors and properties of the AI system itself. Human factors can include the user's education, past experiences, and perception of the technology, while properties include, amongst others, transparency, complexity, and controllability [43].

Trustworthiness, a concept related but distinct from trust, refers to the degree to which an entity or individual is deem worthy of being trusted by a trustor [44]. From a basic point of view, trustworthiness applied to an AI system can be defined as the notion that refers to how users perceive the technology and feel comfortable placing their trust in it. If they feel that they cannot rely on or count on the AI system, thus perceiving it as untrustworthy, they will tend to abandon it and then lose some of its potential benefits. Therefore, while trust is a relational phenomenon, trustworthiness is a characteristic property of the trusted party subject to the perception of the trustee party [31, 44, 45].

In the literature on trust, it is possible to isolate two main tendencies, namely the epistemic and motivational accounts [12].^8^ The epistemic perspective considers trust as a rational choice, where trusting X, an agent, generally involves tasks such as estimating the probability that an agent X will perform a task φ and evaluating the advantages and disadvantages of depending on X to do so. Trust in this framework is understood simply as a ‘reliance property’, that is a concept based on system's performance and delineating (in the medical context) the physician's beliefs and readiness to depend upon the medical AI (e.g., for diagnostic purposes). This dependence is characterised by an absence of deliberate efforts to procure or process additional information concerning the medical AI's competence to formulate a diagnosis, such as through the act of simply monitoring the AI [42]. In this case, motivations and moral obligations seem to play no role in the building of trust. On the contrary, the motivational account introduces non‐epistemic factors in the conceptualisation of trust, allowing for a nuanced distinction between trust and mere reliance. Motivational accounts acknowledge the role of rational assessment, meaning that they still contain some epistemic components of rational assessment, but extend beyond mere reliance and purely epistemic aspects, incorporating additional non‐epistemic elements such as the motivations, intentions, goodwill, and moral obligations of the trustee. As we will see in the next section, we argue that diabetes mHealth apps provide a case where non‐epistemic considerations are unavoidable and show the merits of applying a motivational approach to the concept of trust in the medical AI context.

Another strand of the literature has looked at the fact that, with the presence of artificial agents, the trusted party is a technological artefact and has criticised the attribution of trustworthiness to a digital artefact, in relation to risks of anthropomorphism as the unjustified attribution of human features to nonhuman entities. The idea here is that technological artefacts cannot be objects of trust because they do not possess human characteristics [40, 46, 47, 48]. To deal with this argument, it is possible to isolate two different notions of trust. The first sense of trust is the one amongst humans, also known as interpersonal trust. This type of trust is called ‘H‐H trust’. The second sense of trust involves both humans and AI systems and is named ‘H‐AI trust’. These two types of trust have some commonalities, in particular a shared conceptual core that may contribute to overcoming the limitations of the anthropomorphic objection [12].

To capture this distinction, we can take a closer look at the different fundamental factors characterising those versions of trust. First, H‐H and H‐AI versions of trust share a common conceptual core composed of the following determinants: (i) a basis of reliance, (ii) the adherence to ethical principles, as well as (iii) an analysis of scenarios involving risk and, in particular, vulnerability. The difference between those two types of trust‐related interactions arises from the ethical dimensions themselves. On one hand, goodwill, motivations, interests, and moral obligation are essential factors for H‐H trust, while, on the other hand, human autonomy, transparency, and fairness are pivotal notions for H‐AI trust. TAI systems, akin to trustworthy humans, exhibit reliance, meaning that they aim to consistently produce accurate results, as reliance forms the foundation of trust. However, reliance alone may be insufficient to characterise trust, necessitating additional non‐epistemic elements. Trustworthiness serves as a non‐epistemic guarantee for the trustor, especially in contexts marked by uncertainty and risks where the trustor is vulnerable.^9^ Similarly to H‐H trust, H‐AI trust thus emerges in contexts where the trustee's behaviour is risky and uncertain. It seems natural to assume that the trustworthy characteristic of AI systems is crucial when the technology is used in risky contexts, such as healthcare, where AI decisions can have life‐altering consequences on the trustor, who is often in a position of risk and vulnerability towards the trustee—as we see next in the paper.^10^

## 4 Trustworthy AI and Diabetes mHealth Apps

As discussed in the previous section, according to motivational accounts, reliance is a necessary but insufficient component of trust—every time there is trust, there is reliance too, but not the other way around. To have trust, other elements must be considered in addition to reliance, and these can include risk (intended as the result of hazard, vulnerability, and exposure) as well as ethical values and obligations. Our main point in this section is to show that, while reliance is crucial when evaluating diabetes mHealth apps and assessing their trustworthiness, and indeed this is the standard approach in the literature, it is not enough to determine trustworthiness on its own. We start with a discussion of reliance in the context of diabetes mHealth apps and then move to show that other considerations on risk, ethical values, and obligations should be at the centre of TAI for diabetes mHealth apps.

### 4.1 Reliance

In the context of AI systems, reliance is mainly but not exclusively connected to the performance of the algorithms, such as their accuracy and resilience to attacks. In other words, reliance can be associated to the technical robustness, safety, and accuracy of the system, as well as HCPs' beliefs about the system's performance. In the context of diabetes mHealth apps and their trustworthiness, it is, of course, key that the apps are reliable, and more specifically, this is a crucial starting point to ensure that they are trustworthy. Currently, available diabetes mHealth apps can still encounter technical issues, such as software glitches and crashes, as well as problems related to battery life [24], or can also raise some concerns regarding their design and functionality.^11^ These are clearly technical aspects of the functioning and design of these apps, but as we have seen, reliance is a central component of trustworthiness, and a lack of reliance can lead to significant concerns on the extent to which mHealth apps can be trustworthy. Furthermore, individuals with diabetes depend on support from HCPs to manage their condition, interpret glucose levels, and make informed decisions based on the data [50]. Consequently, the involvement of HCPs is also crucial to effectively support patients in using the app as a nontechnical form of reliance [51].

### 4.2 Risk

There is more to trustworthiness than reliance: it is crucial that the development of diabetes mHealth apps is centred around other considerations and elements if we want to ensure their trustworthiness. Beyond reliance, a first area of focus for these considerations is risk—the assessment of the trustworthiness of diabetes mHealth apps can change and crucially depends on how risky the use of the apps is. We approach risk as resulting from the interplay of hazard, vulnerability, and exposure at both the population and individual levels [52].

Hazard is connected to the potential source of harm. Specifically in the case of diabetes mHealth apps, it may relate, for instance, to any malfunction of the app. Trust is essential between human users and the AI system they interact with, as they may face potential health risks if they do not use it correctly. Patients need to trust their apps because they need their support to accurately regulate their glycaemia; thus, if they blindly follow the measurements of the app that malfunctions, they may be at risk, for example, administering insulin incorrectly. This dependency is more pronounced for certain sub‐populations, such as children, elderly people, and those with low socioeconomic status or cultural barriers. These individuals might lack technology literacy and may be not entirely autonomous in managing their illnesses (in particular, elderly people may suffer from comorbidities), making them more vulnerable [53, 54]. Moreover, patients living in remote locations may not benefit from some auxiliary features of mHealth apps such as the connection with health records and digital educational resources. Here, a crucial way in which risk can be modulated as a condition for trustworthiness has to do with HCPs and their involvement. As highlighted by recent research on the use of diabetes mHealth apps [51, 55], the extent to which individual patients correctly make use of these apps is also dependent on the support of HCPs in guiding their use. This may mitigate patient‐specific risks associated to the management of diabetes and can play a crucial role in supporting the relations between patients, apps, and HCPs and thus enhancing trust.

In addition, the exposure's component of risk defines what could be harmed, in particular, the apps' users. In 2019, there were 46.3 million installations of diabetes mHealth apps worldwide, which corresponds to approximately 11% of patients diagnosed with diabetes globally [20]. Exposure is thus very high, and there are several individuals that run the risk of being harmed using diabetes mHealth apps, in virtue of the numerosity and variability of patients using them, in particular in case of comorbidities and complex medical scenarios.

### 4.3 Ethical Considerations

Beyond reliance and risk dimensions, there are specific ethical considerations that need to be taken into account when developing diabetes mHealth apps in the direction of ensuring trustworthiness and uses of TAI. For instance, according to the European Commission, TAI is characterised as grounded in various ethical principles, including respect for human autonomy and prevention of harm [29], that we consider relevant in the context of diabetes mHealth apps. Regarding autonomy, mHealth apps can promote but also restrict it at the same time [56]. This is because the self‐management in such apps may encourage patients to adhere to a strict clinical regimen rather than promoting their self‐determination [57]. This is especially problematic from a bioethical perspective, as a patient's autonomy in decision‐making may be assisted but cannot *blindly* rely on mHealth indications, potentially resulting in patients over‐trusting medical technologies. However, also deciding what and how much to eat, as well as determining the appropriate insulin dosage, can be challenging when using alternative methods to deal with diabetes care. Again, the extent to which individual users can rely on HCPs and not just apps and devices, if not the possibility of direct data exchange between diabetes mHealth apps and carers, can make a crucial difference here [50, 58].

These apps enable patients to continuously take care of themselves without the need for an HCP, such as a homecare nurse, to assist them. However, many challenges still remain associated with their interactions with patients. Moreover, when the apps do not work (e.g., due to a dead battery, phone updates, or phone being forgotten) or if the algorithm turns out to provide incorrect outcomes, patients may be left on their own and run the risk of not making the right decisions about their treatment, leading to dependency on the apps. This could potentially cause harm if patients do not properly follow their treatment plan. While patients can check their glucose levels by pricking their fingers with a glucose meter, this simply creates dependency on another technology.

Furthermore, it is important to remember that the context where diabetes mHealth apps are applied is a primarily medical context. This calls for the consideration of specific ethical aspects that are particularly significant in medicine and can at least include the normative principles of bioethics: beneficence, non‐maleficence, autonomy, and justice. These principles are standard in contemporary bioethics and show some overlap with previous considerations, but also represent minimal ethical conditions without which medical treatment cannot be considered ethical and trustworthiness can hardly be established.

For instance, adherence to the principles of beneficence and non‐maleficence should clearly be assessed for diabetes mHealth apps. Then, on top of the previous considerations on autonomy, to ensure that diabetes mHealth apps can be the centre of trust by users and patients, we need to make sure that considerations about justice are also addressed. In bioethics, justice is the principle that calls for the respect and promotion of fair, equitable, and appropriate treatment. Justice can thus refer to the fair, equitable, and appropriate distribution of healthcare resources. In the context of diabetes mHealth apps, this can be translated into the need to make sure that access to these apps is fair and equal and does not leave behind some specific social groups characterised by gender, socioeconomic position, and so on. This is a crucial issue in the context of digital approaches to health, and failing to address these considerations can lead to doubt and scepticism towards mHealth apps, reducing their trustworthiness and uptake. Although all the bioethical principles mentioned above may play a role in fostering trust in diabetes mHealth apps, autonomy is particularly essential. Autonomy empowers patients to make informed decisions about their daily diabetes management while retaining control over their personal health data and treatment choices. Users' ability to independently monitor, adjust, and manage their condition through these apps (along with transparency regarding data usage) directly influences both their engagement with the technology and their overall health outcomes.

### 4.4 Potential Objections

When considering the extent to which diabetes mHealth apps can be trusted by users and patients, we argue that we should not forget that trust has medical and ethical implications that can raise deep concerns and modify patient use of the apps.^12^ As we have seen, reliance, adherence to ethical principles, and risk (intended as hazard, vulnerability, and exposure) work together to ensure trust, and the interplay of all these determinants is crucial for trust to fully take place. These determinants are to be jointly considered as necessary conditions for trustworthiness and mHealth apps. This issue of lack of accuracy and, thus, reliance might seriously impact the health of the users. Globally, reliance is just one of the pillars to test when evaluating the notion of trust in mHealth apps because, in this case, it is mainly given by the accuracy of the data. Nonetheless, mere reliance is not enough to ensure trust, and the other determinants play a crucial role too.

One might object that reliance can be enriched with additional characteristics to form a stronger basis for trust and in that case trust would be mainly related to the design of mHealth apps. But this would give rise to the risk of focusing just on the design phase while forgetting other aspects, implying human use and interactions and the different socio‐technical systems in which they may be applied that are crucial in diabetes treatment. Therefore, for diabetes mHealth apps, we can claim that reliance alone cannot ensure trust. Consequently, a stronger notion of reliance interpretable as a substitute for trust cannot be applied to diabetes mHealth apps. Indeed, reliance and trust should be distinguished to enable a comprehensive assessment of the apps throughout all phases of their life cycle, encompassing both technical and ethical evaluations.

## 5 Conclusions

The digital transformation of healthcare systems through the integration of mHealth apps based on AI algorithms is revolutionising patient care, particularly in the realm of chronic disease management, such as diabetes. These AI‐powered mHealth apps provide patients the opportunity to monitor their health conditions remotely and continuously, thereby enabling them to manage their own well‐being by providing personalised information based on various data.

While the potential benefits of mHealth apps are significant, concerns have been raised regarding trust and trustworthiness towards their AI algorithms. Trust in AI is a multifaceted notion that encompasses aspects such as reliance, risk, as well as ethical principles. Patients must be able to trust the accuracy and completeness of the data generated by the AI algorithm of the apps, as well as the treatment's recommendations made based on these data. In addition, patients may be vulnerable due to various factors, such as age, health literacy, geographic location, and access to HCPs, which can impact their dependency on their apps.

Ethical considerations play a crucial role in the development and deployment of AI‐powered mHealth apps. For instance, the European Commission emphasises the importance of ethical principles, such as respect for human autonomy and prevention of harm in the context of TAI. While mHealth apps have the potential to promote patient autonomy by enabling self‐care and reducing reliance on HCPs, there is also a risk of over‐dependence and misuse of these technologies. Patients might face harm if they are unable to make informed decisions about their treatment in the absence of functioning AI algorithms or technical issues.

To address these challenges, a comprehensive approach to building trust in AI‐powered mHealth apps is essential. This involves ensuring not only the reliance and accuracy of AI algorithms but also addressing potential risks and vulnerabilities and upholding ethical principles such as autonomy and prevention of harm together with other classical bioethical values. Indeed, while reliance on these technologies is necessary, it is insufficient for establishing trust. Trust encompasses additional elements such as risk, which can be further analysed as hazard, vulnerability, and exposure, as well as ethical values and obligations. We have emphasised that reliance is closely tied to the performance of the AI system, including its accuracy and resilience against potential attacks. We have highlighted existing technical issues within current diabetes mHealth apps, such as software glitches, user interface challenges, and integration problems with other medical devices, which can undermine user trust. Finally, we have highlighted the key role that HCPs can play in shaping mHealth diabetes apps into trustworthy medical technologies.

## Author Contributions

All authors contributed equally. In particular, Sophie Materne supervised the project and wrote Section 2, Daniele Chiffi wrote Section 3, and Stefano Canali wrote Section 4. All authors reviewed the final manuscript.

## Conflicts of Interest

The authors declare no conflicts of interest.

## Data Availability

Data sharing not applicable to this article as no datasets were generated or analysed during the current study.

## Endnotes

1 The literature reports several definitions of AI. According to the Organisation for Economic Co‐operation and Development's [1] definition that we follow in this paper, an AI system is ‘machine‐based system that, for explicit or implicit objectives, infers, from the input it receives, how to generate outputs such as predictions, content, recommendations, or decisions that can influence physical or virtual environments. Different AI systems vary in their levels of autonomy and adaptiveness after deployment’. This definition aligns well with recent philosophical work, such as the revisited legal definition suggested by Floridi: ‘artificial intelligence refers to an engineered system that can, for a given set of human‐defined objectives, generate outputs—such as content, predictions, recommendations, or decisions—learn from historical data, improve its own behaviour, and influence people and environments' [2].

2 Nonetheless, it is of high importance to remember that diabetes mHealth apps do not substitute HCPs, but rather assist them in monitoring their health status and condition progression by efficiently supporting the whole treatment process.

3 See: https://camdiab.com/fr-ch.

4 See: https://esysta-diabetes.com/.

5 See: https://www.dexcom.com/apps.

6 The AI‐Act is a regulatory framework that clearly differentiates systems based on their varying levels of risk.

7 Even though their widespread adoption within society still depends on their potential users' willingness to interact and trust them [35].

8 This is a general and not an exhaustive distinction and we do not want to say that all accounts of trust directly fall into one of these two categories.

9 On uncertainty and technological innovation, see Chiffi et al. [49].

10 For example, regarding AI technologies based on machine learning (ML) algorithms, there is a potential lack of interpretability, thus undermining the patient's trust in the medical field.

11 It is worth noting that overreliance over diabetes mHealth apps also presents a non‐negligible risk.

12 The distinction between the intended use of mHealth devices and their actual use may present many ethical problems [59].
